# Magnetic, conductive nanoparticles as building blocks for steerable micropillar-structured anodic biofilms

**DOI:** 10.1016/j.bioflm.2024.100226

**Published:** 2024-10-03

**Authors:** René Wurst, Edina Klein, Johannes Gescher

**Affiliations:** Institute of Technical Microbiology, University of Technology Hamburg, Hamburg, Germany

## Abstract

In bioelectrochemical systems (BES), biofilm formation and architecture are of crucial importance, especially for flow-through applications. The interface between electroactive microorganisms and the electrode surface plays an important and often limiting role, as the available surface area influences current generation, especially for poor biofilm forming organisms. To overcome the limitation of the available electrode surface, nanoparticles (NPs) with a magnetic iron core and a conductive, hydrophobic carbon shell were used as building blocks to form conductive, magnetic micropillars on the anode surface. The formation of this dynamic three-dimensional electrode architecture was monitored and quantified *in situ* using optical coherence tomography (OCT) in conjunction with microfluidic BES systems. By cyclic voltammetry the assembled three-dimensional anode extensions were found to be electrically conductive and increased the available electroactive surface area. The NPs were used as controllable carriers for the electroactive model organisms *Shewanella oneidensis* and *Geobacter sulfurreducens*, resulting in a 5-fold increase in steady-state current density for *S. oneidensis*, which could be increased 22-fold when combined with Poly(3,4-ethylenedioxythiophene)-poly(styrenesulfonate) (PEDOT:PSS) aggregates. In the case of *G. sulfurreducens*, the steady-state current density was not increased, but was achieved four times faster. The study presents a controllable, scalable and easy-to-use method to increase the electrode surface area in existing BES by applying a magnetic field and adding conductive magnetic NPs. These findings can most likely also be transferred to other electroactive microorganisms.

## 1 Introduction

Some microorganisms can use solid-state anodes in bioelectrochemical systems (BES) as the terminal electron acceptor of their respiratory chain. The oxidation of an organic or inorganic substrate is therefore associated with the transfer of respiratory electrons to the anode of an electrochemical circuit. This energy conversion process can be used in a variety of applications, e.g. in wastewater treatment or in anode-assisted fermentation processes [[1], [2], [3], [4], [5]].

The electron transfer reaction can be catalyzed directly by the physical contact of cells or conducting cell appendages with the electrode or by electron shuttles produced by the organisms [[6], [7], [8], [9], [10], [11], [12]]. Direct electron transfer via cells or cell appendages appears to be the most efficient route for electron transfer in these systems [[13], [14], [15], [16], [17], [18], [19]]. So far, *Shewanella oneidensis* and *Geobacter sulfurreducens* are the best understood model organisms for anode reduction. *G. sulfurreducens* can form multilayer conductive biofilm structures on anodes, and the current densities that can be achieved with this organism are significantly higher than with *S. oneidensis* [[20], [21], [22]]. The latter organism tends to form thin and often single-layered biofilms on anode materials [23]. This could be due to the fact that it is not able to form nanowires (as shown in *G. sulfurreducens*) that can conductively connect the cells to each other and to the electrode [15,16,[24], [25], [26], [27], [28], [29], [30]]. Apart from this ability of nanowire formation, both strains are quite similar in terms of their electron transfer strategy. Both rely on a network of multi-heme *c*-type cytochromes that interact conductively with each other and transport electrons from the cytoplasmic membrane to the cell surface. The last step is also carried out via multi-heme *c*-type cytochromes. These cytochromes are rather non-specific, and electron transfer takes place on surfaces characterized by their redox potential, which must be about −200 mV relative to a standard hydrogen electrode or higher [25,27,[29], [30], [31], [32], [33], [34], [35]].

This lack of specificity in electron transfer has enabled several strategies to increase current densities in bioelectrochemical systems by improving the cell-electrode interface and/or developing more conductive cells [[36], [37], [38], [39], [40], [41], [42], [43], [44], [45]]. For example, the surface of *S. oneidensis* cells was functionalized with Fe_3_O_4_ or gold NPs to increase cellular conductivity. These functionalized cells were characterized in bioelectrochemical batch systems and higher current densities were achieved. This effect could be promoted by combining both strategies, so that gold-functionalized cells were connected to Fe_3_O_4_ NPs, which were then used to attract the cells to a magnetic anode [42]. A similar approach with *n-type* doped Fe_3_O_4_/carbon composite-NPs was also performed with *Geobacter* cells [43,44]. Functionalization of *Shewanella* cells with carbon dots [41] and silver NPs [36] was also performed and again the current density could be increased. Overall, the maximum increase in current density reported for *Shewanella* by using these nanoparticle-based strategies was achieved by using silver NPs, resulting in approximately 8-fold higher maximum current densities [36].

All these strategies mentioned above lead not only to an increased electron transfer rate of the biocatalysts, but also to an increase in the anodic surface area, as the cell/nanoparticle agglomerates are conductively connected to the electrode, and it has not yet been shown to what extent the increase in surface area alone could probably affect the quantified current densities. Furthermore, in the case of magnetic particles or the reported increase in biofilm formation due to silver NP formation, no attempt has been made to map the resulting electrode architecture or defining its stability. Furthermore, all studies reported so far unfortunately originate from batch and not continuously operated systems. The results from these two types of reactors will most probably differ due to the continuous dilution of endogenously produced shuttle molecules that would accumulate in batch systems. However, future applications are likely to be operated in continuous mode using low-cost dilute carbon sources.

This study was conducted to answer two research questions. First, we aimed to understand how we could build dynamic three-dimensional electrode structures based on the interaction of magnetic NPs with a magnetic anode electrode. To answer this question, we used a recently introduced microfluidic imaging platform [22] and observed the continuous evolution of three-dimensional structures based on NPs using OCT. Quantitative image analysis and electrochemical measurements were used to estimate the active anode area. Subsequently, we aimed to understand how these abiotic conductive pili can be used as electron acceptors for *S. oneidensis* and *G. sulfurreducens* biofilms and how functionalization and synthetic extracellular polymeric structures can influence electroactivity. Our strategy led to a 22-fold increase in steady-state current density for *S. oneidensis*, while current generation by *Geobacter* cells could only be accelerated four-fold but not increased.

## 2 Materials and methods

### 2.1 Strains and growth conditions

*Shewanella oneidensis* MR-1 wild type [46] and *Geobacter sulfurreducens* PCA wild type [47] were cultivated and washed as described by Klein et al. [22]. For experiments with NPs and cells, the corresponding NP suspension (see below) was freshly prepared and added to cell suspensions with an optical density of 2.0 at 600 nm with a NP concentration of 100 μg ml^−1^ prior to inoculation. Control experiments without NP addition were conducted with the same optical density. Inoculation was carried out as described by Klein et al. [22]. For all experiments, cells were resuspended in minimal medium according to Dolch et al. [48]. In experiments where PEDOT:PSS aggregates were formed additionally, the minimal medium was supplemented with 40 mM fumarate. All experiments were conducted at a temperature of 30 °C.

### 2.2 Flow cell system

**Fig. 1:**
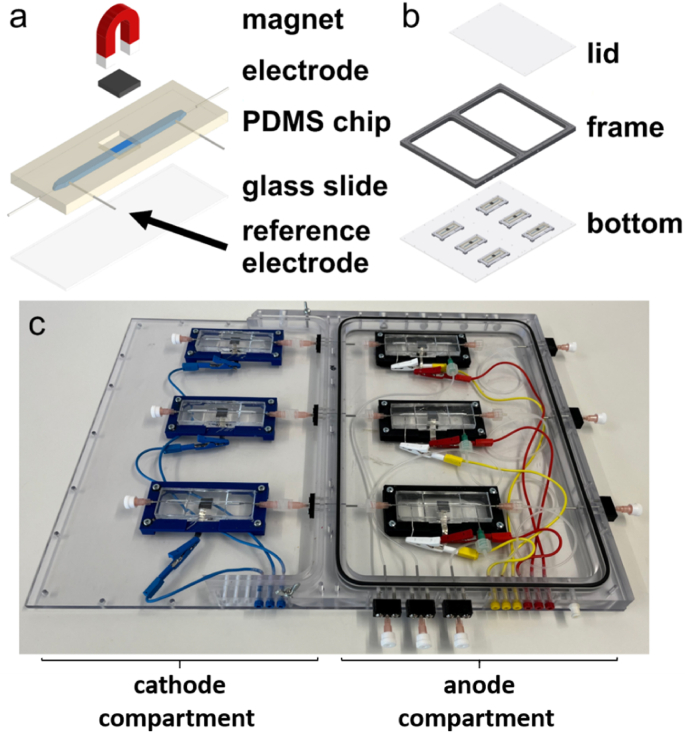
**Platform for electroactive biofilm cultivation and analysis.** The microfluidic cultivation chips (**a**) are composed of polydimethylsiloxane (PDMS) and the channel is sealed by plasma bonding to a glass slide. A 1 × 1 cm graphite electrode and a 1 × 1 cm neodymium N52 block magnet were integrated in the center, additional access points allow fluidic access via insertion of cannulas, as well as the addition of the tailored reference electrode. Three sets of two microfluidic reactors (anode and cathode), are placed in a chamber (**b**), made up of a bottom plate, frame and lid. The lid is positioned on the anodic compartment to allow for anoxic cultivation by purging with 80 % N_2_/20 % CO_2_ gas. A photograph of the frame can be seen in **c**. This setup and figure are based on the platform presented by Klein et al. [22] and slightly modified for the assembly of magnetic micropillars.

Bioelectrochemical experiments were conducted, based on a recently developed microfluidic cultivation platform [22]. Graphite composite electrodes (PPG 86, Eisenhuth GmbH & Co. KG, Osterode am Harz, Germany) with an effective surface area of 3 × 10 mm were integrated into the cultivation channel. To form a BES two reactors were connected. In this setup the upstream reactor contained the working electrode and carried a customized Ag/AgCl reference electrode downstream of the working electrode. Each reactor has a volume of 354 μl, resulting in a hydraulic retention time of 5.3 min with a flow rate of 4 ml h^−1^. Additionally, a neodymium N52 block magnet (Webcraft GmbH, Gottmadingen, Germany) with the dimensions 10× 10 × 1.2 mm was placed directly behind each working electrode (Fig. 1). The anode compartment was kept under anoxic conditions by purging with 80 % N_2_/20 % CO_2_ gas, while the cathode compartment was kept under oxic conditions. The electrodes were connected to a potentiostat (BioLogic VMP-300, Seyssinet-Pariset, France) via a silver foil (0.1 mm; Chempur, Karlsruhe, Germany). The anode potential was set to 0 V (against SHE) and the current was monitored for at least 40 h after inoculation.

### 2.3 Nanoparticle pretreatment and incubation with bacteria

To facilitate dispersion in aqueous solutions the NPs first had to be hydrophilized. 10 μg of sterile iron nanopowder with hydrophobic carbon shell (>83 % Fe, <14 % C, actual particle size <60 nm; PlasmaChem GmbH, Berlin, Germany) were added to 1 ml sterile Poly(3,4-ethylendioxythiophen)-poly(styrolsulfonat) (PEDOT:PSS, 1.5 % in H_2_O, neutral pH, high-conductivity grade; Merck KGaA, Darmstadt, Germany). Subsequently, the mixture was treated in an ultrasonic bath for 10 min. The resulting suspension was directly added to a cell suspension (see above).

### 2.4 Live/dead cell imaging

For live/dead staining of cells attached to NP agglomerates, 1.5 μl propidium iodide (20 mM in dimethyl sulfoxide) and 1 μl Syto9 solution (5 mM in ddH_2_O; both from Invitrogen AG, Carlsbad, CA, US) were added to 1 ml of a freshly prepared cell-nanoparticle suspension in minimal medium (as described above) within a 1.5 ml centrifuge tube. The reaction mixture was kept in the dark for 20 min. All subsequent steps were also performed under exclusion of light. The sample was placed in a magnetic separation rack and washed twice with 1 ml phosphate buffered saline (PBS; 136.9 mM NaCl, 2.7 mM KCl, 10.1 mM Na_2_HPO_4_, 1.8 mM KH_2_PO_4_) to remove excess staining solution and unbound cells. 10 μl of the suspension were transferred onto a microscope slide and covered with a glass coverslip. Microscopic imaging was performed using a Leica DM5500B microscope, a Leica K5-14401820 camera, and Leica Application Suite X software version 3.8.0.26413 (Leica Microsystems, Wetzlar, Germany). Images of the nanoparticle-cell agglomerates were taken with the N PLAN 100x/1.25 oil objective (Leica Microsystems, Wetzlar, Germany). To assess the NPs potential cytotoxicity towards the bacterial strains, the amount of metabolically active (live) cells within the agglomerates was compared to the cells with damaged membrane (dead cells). The percentage of live and dead cells was determined using (Fiji Is Just) ImageJ version 2.1.0/1.53 [49]. Therefore, the images were converted to 8-bit-images (2^8^), binarized with an appropriate threshold (0–255) and the percentages of live and dead cells were determined using Formula 1 and 2, respectively. An abiotic control experiment did not show any significant background fluorescence of the NPs by Syto9 or propidium iodide staining.

Formula 1 $${percentage}_{live}=\frac{{pixels}_{live}}{{pixels}_{live}+{pixels}_{dead}}\times 100$$

Formula 2 $${percentage}_{dead}=\frac{{pixels}_{dead}}{{pixels}_{live}+{pixels}_{dead}}\times 100$$

### 2.5 Growth experiments

Growth curves of *S. oneidensis* were performed using an Infinite200Pro plate reader (Tecan Trading AG, Switzerland) until stationary phase was reached. Precultures were grown overnight in LB at 30 °C and 180 rpm. Experiments were performed in quadruplicates in a 12-well plate in the presence and absence of 100 μg ml^−1^ PEDOT:PSS functionalized NPs in the growth medium at 30 °C and 180 rpm. Optical density at a wavelength of 600 nm (OD_600_) was measured in a 15-min interval, with a starting OD_600_ value of 0.03. The wells were filled with 2 ml of medium each.

### 2.6 Optical coherence tomography (OCT) and determination of active electrode surface area

Mesoscopic structures of abiotic NPs aligned in the magnetic field on the working electrode were captured via optical coherence tomography (OCT), using a Ganymede™ spectral domain system (GAN611C1-SP1, Thorlabs GmbH, Dachau, Germany). Parameters were set as described by Bauer et al. [50] and datasets were processed with (Fiji Is Just) ImageJ version 2.1.0/1.53 [49]. Height maps of the NP decorated electrodes were generated based on the process routine developed by Wagner and Horn [51]. Surface coverage and mean height of the micropillars on the anode surface were determined via these height maps. To assess their mean diameter, 100 micropillar diameters were measured via (Fiji Is Just) ImageJ. With the information on surface coverage, mean diameter and mean height, the active electrode surface area of the micropillar-structured anode was calculated. Hereby, it was assumed that each micropillar has cylindrical shape.

### 2.7 Determination of active electrode surface area via cyclic voltammetry

Electroactive surface area of the NP decorated electrode was determined electrochemically via the cyclic voltammetry (CV) peak current method as described by Zhu and Zhao [52], additionally to the graphical analysis of the OCT images.

For the CV peak current method, the three-electrode microfluidic flow cell setup described above was employed. 5 mM K_4_[Fe(CN)_6_)] in 1 M aqueous KOH solution served as electrolyte. On the working electrode surface the oxidation reaction of ferrocyanide is:

Formula 3 $${\left[\text{Fe}\left(\text{CN}\right)6)\right]}^{4-}\to {\left[\text{Fe}\left(\text{CN}\right)6)\right]}^{3-}+e^{-}$$

The reaction is controlled by diffusion of ferrocyanide ions and has good reversibility on carbon electrodes [53]. Thus, the peak current is directly proportional to the electroactive surface area of the working electrode, as expressed by the Randles-Sevcik equation:

Formula 4 $$I_{p}=268600n^{\frac{3}{2}}AD^{\frac{1}{2}}C\nu^{\frac{1}{2}}$$

*I*_*p*_ (A) represents the peak current, *n* is the number of electrons transferred in the redox event (here n = 1), *A* (cm^2^) is the electroactive surface area, *D* (cm^2^ s^−1^) is the diffusion coefficient (6 x 10^−6^ for [Fe(CN)_6_)]^4-^ [54]), *C* (mol cm^−3^) is the concentration of the redox active species (here 5 x 10^−6^ for [Fe(CN)_6_)]^4-^) and *ν* (V s^−1^) constitutes the scan rate. CVs were conducted with scan rates of 0.01 V s^−1^.

### 2.8 Sample preparation for scanning electron microscopy (SEM)

After 40 h of anodic cultivation in the microfluidic reactor, a *G. sulfurreducens* biofilm with embedded NPs was first fixed in the reactor with a 4 % formaldehyde in PBS solution. Fixation was performed under continuous flow conditions of 4 ml h^−1^ for 1 h, followed by stepwise desalting with absolute ethanol. Subsequently, the biofilm-nanoparticle hybrid-bearing anode was carefully lifted out of the reactor and directly transferred into a high-pressure autoclave with a total volume of 3.9 l for supercritical drying with CO_2_. Drying was carried out at 40 °C and 120 bar. A continuous stream of CO_2_ (flow rate = 80–110 g min^−1^) was passed through the autoclave until complete extraction of the ethanol was achieved after 1 h. The dried sample was collected after slow depressurization of the autoclave and stored in a sealed tube in a desiccator until SEM analysis. The sample was sputtered with gold and analyzed with a Zeiss Supra 55 VP SEM.

## 3 Results

### 3.1 Controlled assembly of magnetic micropillars on an anode surface via conductive and magnetic nanoparticles

**Fig. 2:**
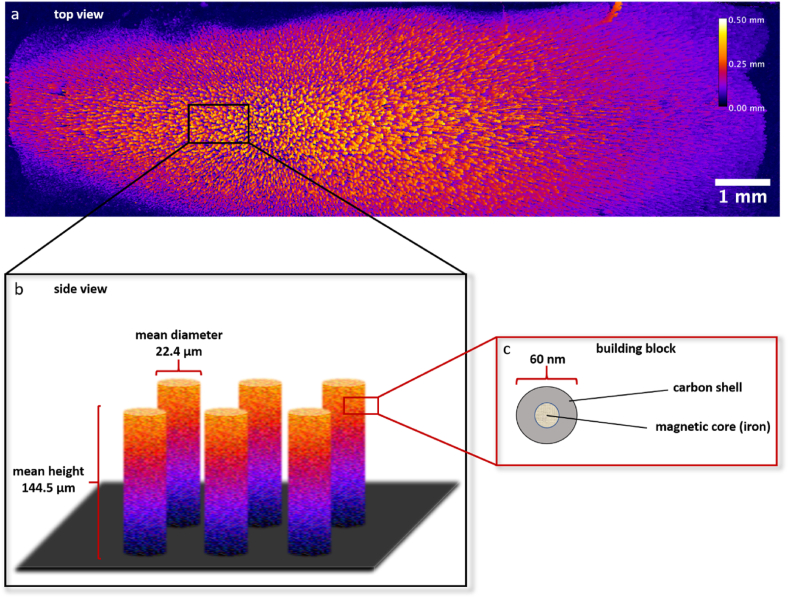
**Height map (a) and schematic illustration of conductive, magnetic micropillars (b) on the anode surface and their corresponding nanoparticle building blocks (c).** The height map (**a**) provides a top view of the anode surface, decorated with micropillars throughout. The image was generated via OCT measurement and subsequent data processing with Fiji (Is Just ImageJ). A schematic illustration (**b**) shows a zoomed in side view of several micropillars on the anode surface, along with their calculated mean diameter and height. This illustration is simplified for clarification reasons and shows a higher regularity than the height map. In **c** the single NP building block of the assembled micropillars is illustrated. It consists of a magnetic iron core and a hydrophobic carbon shell.

To facilitate the formation of a steerable three-dimensional conductive structure on a flat electrode area, magnetic NPs with an iron core and a hydrophobic carbon shell were chosen as building blocks (Fig. 2c). The carbon shell offers the advantage of being chemically inert while also electrically conductive. Still, the hydrophobicity of the particles necessitated a coating with PEDOT:PSS to allow for controllable handling in aqueous solutions. The latter polymer is amphiphilic and consequently enabling dispersion in aqueous media. Moreover, PEDOT:PSS is biocompatible and conductive [[55], [56], [57], [58], [59]]. The iron core allows for a controlled assembly of 3D structures in a desired location, since the NPs will only gather in the presence of an externally applied magnetic field.

To assess the dimensions of the extended electrode area, the NPs suspension was injected for 2 h with a flow rate of 2 ml h^−1^ into the flow cell system, carrying a neodymium N52 block magnet below the anode (Fig. 1). The injected NPs were attracted by the magnetic field, leading to the formation of micropillars throughout the anode surface (supplementary video 1). Subsequently, the electrode surface was scanned via OCT and a height map was created (Fig. 2a). This height map was used to determine the surface coverage of the micropillars, resulting in a value of 42.5 % of the overall surface area being covered. The height map was also employed to determine the mean height and diameter of the micropillars, resulting in a height of 144.5 ± 82.4 μm and a diameter of 22.4 ± 6.6 μm (Fig. 2b schematically displays a side view). With these values it was possible to determine the overall mean electroactive surface area of the electrode, by assuming a cylindrical shape of the micropillars, resulting in a value of 3.6 cm^2^, or an increase in surface area by factor 12, respectively.

### 3.2 Advances in steered removal of micropillars from anode surface

Following the successful assembly of magnetic micropillars on the anode surface, efforts have been made to enable the controlled movement of the NPs within the reactor. Hence, a movable magnet holder was designed allowing to move the magnet below the anode structure within the anoxic chamber of the BES (supplementary video 2). First, micropillars were formed as previously described with the magnet positioned underneath the anode as conducted before. Then, the magnet was moved downstream to the side of the anode as far as the positioning within the frame allowed. The magnetic nanoparticles exhibited only slight movement, even when the magnet was moved back and forth several times (supplementary video 3). This observation suggests that the infrastructure requires larger anoxic chambers within the frames to position larger permanent magnets further away from the anode, enabling significant movement of the nanoparticles.

Nevertheless, an alternative approach was also tested to show that disassembly of the structures is possible. Hence, a second, stronger magnet (neodymium N42 block magnet 10× 10 × 5 mm; Webcraft GmbH, Gottmadingen, Germany) was positioned on the top side of the microfluidic channel, opposite to the first magnet that was used to assemble the micropillars initially (supplementary video 4). Once the second magnet was in place, the OCT could not be utilized for imaging because of shielding from the magnet, thereby limiting observations to those made before the magnet was added and after it was removed. Nevertheless, it is evident that upon removal of the top magnet, the NPs are drawn back onto the anode surface, where they reorganize into micropillar structures. This approach demonstrates, that it is possible to steer and direct the NPs not only onto the anode surface but also away from it and back onto it. However, it requires direct access to the anode reactor without the frame lid (Fig. 1), as the distance for the top magnet to the anode would otherwise be too far. This limitation prevents the current setup from being used for biotic experiments, as the lack of a lid obstructs the establishment of an anoxic environment. As a result, we opted to conduct experiments using a non-movable permanent magnet (Fig. 1) to maintain controlled conditions.

### 3.3 Electrochemical assessment of surface enhancement

**Fig. 3:**
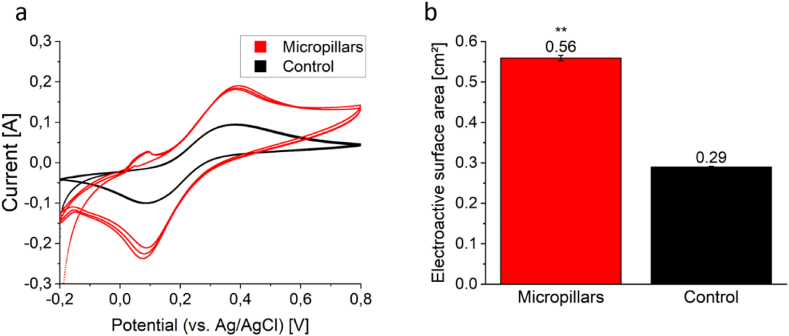
**Electrochemical assessment of surface enhancement.** Cyclic voltammetry of untreated anode surface (black) and microstructured anode surface (red, **a**). By retrieving the values for peak current I_p_ it is possible to calculate the electroactive surface area via Randles-Sevcik equation (**b**). The error bars represent the standard deviation in I_p_ from three cyclic voltammetry cycles. Asterisks represent significant differences (unpaired *t*-test: ∗∗ = p < 0.01). (For interpretation of the references to color in this figure legend, the reader is referred to the Web version of this article.)

In addition to the graphical analysis via OCT, an electrochemical evaluation of the micropillar-structured anode surface was performed. First, a CV of the untreated 2D anode surface was performed, resulting in a peak current I_p_ of 0.094 ± 0.001 A. This was followed by the assembly of the magnetic micropillars and a subsequent CV of the 3D anode, resulting in a peak current I_p_ of 0.19 ± 0.0045 A. Both graphs are shown in Fig. 3a. The peak current values can be used to calculate the electroactive surface area using the Randles-Sevcik equation, leading to an electroactive surface area of 0.29 ± 0.0022 cm^2^ for the untreated anode and 0.56 ± 0.0065 cm^2^ for the micropillar-structured anode, respectively (Fig. 3b). This indicates, that the micropillars formed by magnetic NPs increase the electroactive surface area by at least a factor of 2.

### 3.4 Electroactive cells adhere onto nanoparticle aggregates without loss of viability

**Fig. 4:**
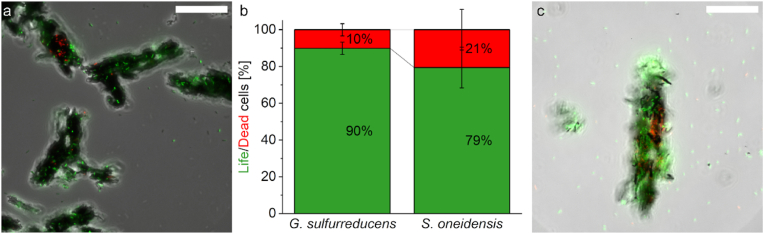
**Life/Dead staining of *G. sulfurreducens* (a) and *S. oneidensis* (c) cells on agglomerated magnetic, conductive nanoparticles**. In **b** the percentage distribution of living and dead cells is shown, **a** and **c** represent microscopic images of living (green) and dead (red) cells in an overlay image. The white bar in **a** and **c** indicates a length of 20 μm. The error bars in **b** represent the standard deviation of technical triplicates. (For interpretation of the references to color in this figure legend, the reader is referred to the Web version of this article.)

After successfully extending the electroactive anode surface with conductive, magnetic micropillars, the NPs were investigated regarding their suitability as carriers for the electroactive model organisms *S. oneidensis* and *G. sulfurreducens*. Therefore, a cell viability assay was performed to quantify possible cytotoxic effects of the NPs. As visible in Fig. 4, the particles do not seem to have a severe negative impact on cell viability, as for *G. sulfurreducens* 90 ± 3 % and for *S. oneidensis* 79 ± 11 % of the cells attached to the NP agglomerates are living. Furthermore, growth curves of *S. oneidensis* were generated in the presence and absence of PEDOT:PSS functionalized NPs in the growth medium. The experiments showed no significant differences in growth behavior with μ_max_ values of 0.24 ± 0.01 h^−1^ for *S. oneidensis* without NPs and 0.23 ± 0.03 h^−1^ for *S. oneidensis* with NPs, respectively (data not shown, experiments were performed in quadruplicates, unpaired *t*-test: p > 0.5). Hence, the NPs are not only suitable as an electrode surface extension in abiotic setups, but also for BES. Beyond that, the NP aggregates can function as steerable carriers to direct cells to a desired location as e.g. an anode and do not hinder cell growth.

### 3.5 Conductive, magnetic nanoparticles improve performance of BES

**Fig. 5:**
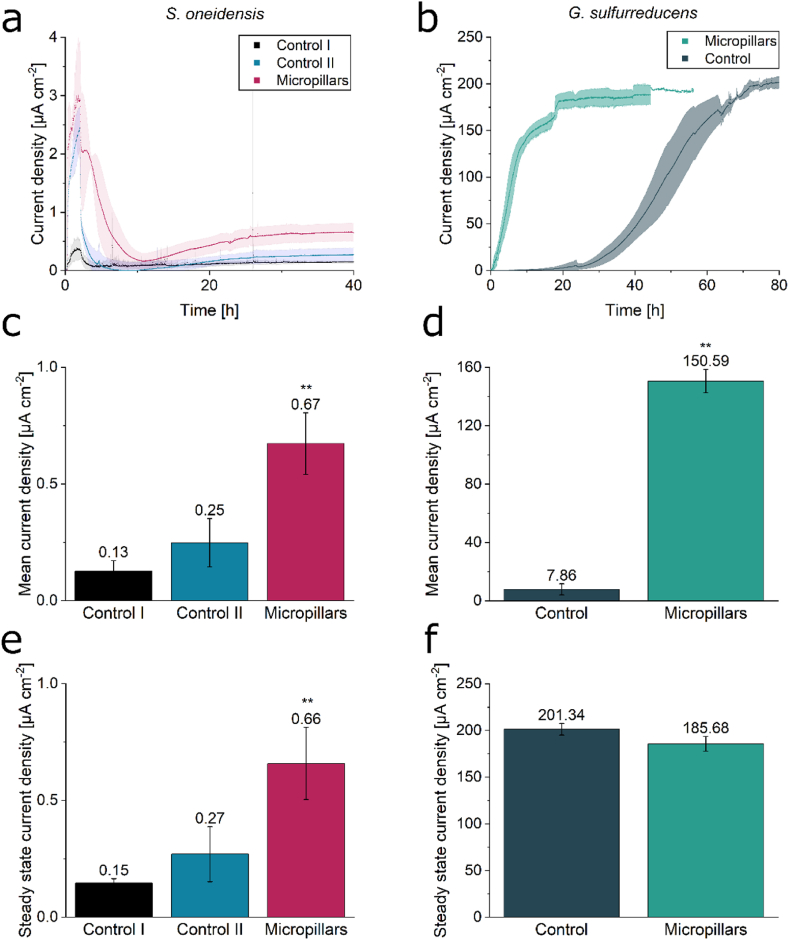
**Comparison of current density with and without the use of conductive, magnetic micropillars for *S. oneidensis* and *G. sulfurreducens*.** In panels **a**, **c**, and **e**, the current densities for *S. oneidensis* are shown for three conditions: without NP addition (Control I), with NP addition but without a magnet placed beneath the anode (Control II), and with NP addition and a magnet positioned below the anode (Micropillars). In panels b, d, and f, the current densities for *G. sulfurreducens* are presented for two conditions: without NP addition (Control) and with NP addition along with a magnet placed below the anode (Micropillars). **a** and **b** show the current density over time of *S. oneidensis* and *G. sulfurreducens*, respectively. In **a** the inoculation time (first 2 h) is indicated with a grey box. In **c** and **d** the mean current densities after 40 h of both microorganisms are plotted, while **e** and **f** show the steady state current densities. Error bars represent the standard deviation from individual triplicates and in case of Control II from individual duplicates. Asterisks represent significant differences towards Control I and Control, respectively (unpaired *t*-test: ∗∗ = p < 0.01).

The utilization of NP aggregates as steerable carriers in BES was tested with the electroactive model organisms *S. oneidensis* and *G. sulfurreducens*. In both cases the NP suspension was mixed with the cell suspension directly before inoculation. First, the current density produced by *S. oneidensis* without the addition of nanoparticles (Control I) was compared to that with nanoparticles added but without a magnet positioned beneath the anode (Control II). This was further compared to an experiment involving both the addition of nanoparticles and the presence of a magnet (Fig. 5a–c, e). Current density on the micropillar-structured anode was higher throughout the experiment (40 h) with a steady-state current density significantly higher compared to Control I with a regular two-dimensional anode, resulting in a mean current density of 0.67 ± 0.13 μA cm^−2^, compared to a mean current density of 0.13 ± 0.046 μA cm^−2^ for the Control I experiment, while the addition of NPs without a magnet beneath the anode (Control II) seems to have a slight positive effect on mean current density values (0.25 ± 0.10 μA cm^−2^) but no significant increase compared to Control I (unpaired *t*-test: p > 0.1). It is also noteworthy, that for *S. oneidensis* the peak current is reached at the end of the inoculation (2 h, indicated with grey box in Fig. 5a). Since *S. oneidensis* is a rather poor anodic biofilm-former and reaches comparatively low current densities, this peak stems most probably from the flushed-through planktonic cells that depend on the anode as electron sink. Over time the planktonic cells are washed out with the medium flow. The more complex three-dimensional micropillar structured anode seems to retain planktonic cells for a longer time compared to the 2D anode of the control experiments. This leads to more planktonic cells utilizing the anode as electron sink for a longer time. Eventually, after 10 h, the planktonic cells are also washed out in the 3D anode setup and from that point on the remaining cells adhered onto the anode are left to transfer electrons towards the electrode and form a biofilm. A different behavior was observed with *G. sulfurreducens*. Here, the micropillars did not lead to a significant change in the steady-state current density (unpaired *t*-test: p > 0.05), but the start-up time was drastically shortened, so that the plateau phase was reached after 18 h, compared to 72 h in the control experiment. Hence, a 4-fold increase in startup time was reached. This also has a significant effect on the mean current density after 40 h, where the micropillar-structured anode reached values of 150.59 ± 8.02 μA cm^−2^, compared to a mean current density of 7.86 ± 3.77 μA cm^−2^ for the control experiment (Fig. 5b–d, f).

**Fig. 6:**
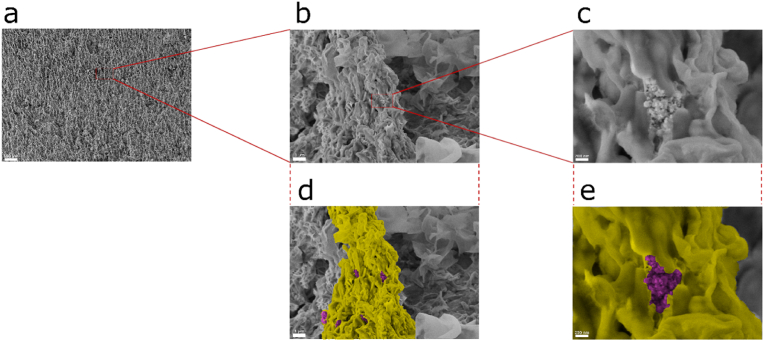
**SEM images of anodic, micropillar-structured *G. sulfurreducens* biofilm.** Overview, with a multitude of micropillars visible (**a**), focus on one micropillar (**b**,**d**) and focus on several NPs that are not covered by biofilm as building blocks of each micropillar (**c**,**e**). The majority of the micropillar is covered with biofilm and single *G. sulfurreducens* cells are visible. For better visibility the biofilm matrix on the micropillar is colored yellow and the NPs are colored magenta in **d** and **e**. (For interpretation of the references to color in this figure legend, the reader is referred to the Web version of this article.)

Fig. 6 displays SEM images of a *G. sulfurreducens* biofilm grown on a micropillar-structured anode. Although the necessary sample pretreatment for electron microscopy will likely change the biofilm structure, it is evident that the majority of the micropillar is covered with biofilm (yellow) and only minor exceptions without coverage (magenta) are visible, indicating that the cells are able to form biofilms along the whole micropillar structures.

### 3.6 Combination of micropillars with PEDOT:PSS aggregates further improves the performance of *S. oneidensis*

**Fig. 7:**
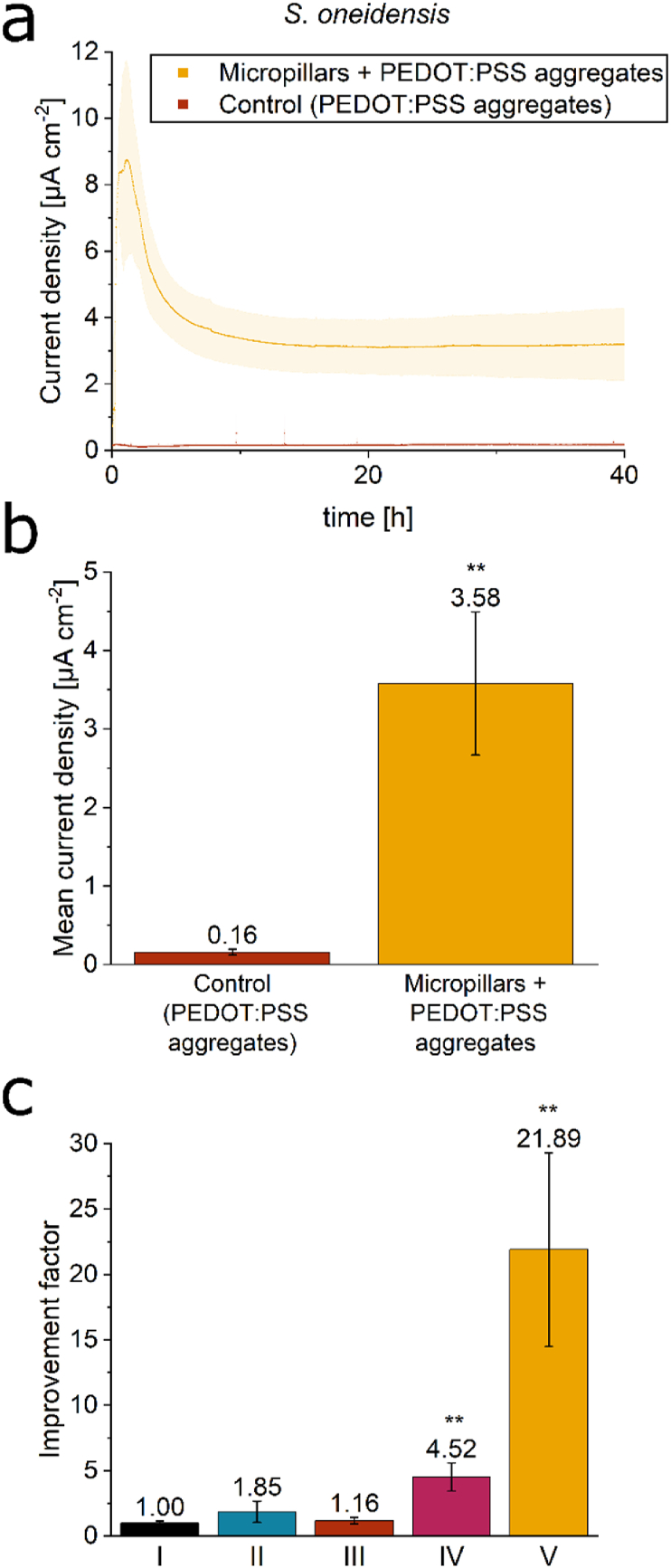
**Combination of micropillars with PEDOT:PSS aggregates further improves current density of *S. oneidensis*. a** shows the current density over time for the addition of only PEDOT:PSS aggregates as well as for the addition of a combination of magnetic, conductive NPs and PEDOT:PSS aggregates, while in **b** the corresponding mean current densities after 40 h can be seen. Additionally, the improvement factors in steady-state current density compared to Control I (no NP addition; I) are shown for Control II (NP addition without a magnet; II), for the addition of only PEDOT:PSS aggregates (no NP addition; III), for the addition of only magnetic, conductive nanoparticles (IV), and for the combination of nanoparticles and PEDOT:PSS aggregates (V) (see panel c). Error bars indicate the standard deviation from individual triplicates, and for Control II, from individual duplicates. Asterisks denote significant differences compared to the Control I experiment (unpaired *t*-test: ∗∗ = p < 0.01).

To investigate whether the current density production of *S. oneidensis* can be improved by further increasing the conductive surface area, a mixture of conductive magnetic NPs, PEDOT:PSS aggregates and cell suspension was prepared directly prior to inoculation in the BES. The PEDOT:PSS aggregates, which formed spontaneously in the preculture in the presence of fumarate, served as a synthetic, conductive biofilm matrix for *S. oneidensis*, which could become entangled in the micropillars on the anode surface. As shown in Fig. 7a and b the current density production was significantly increased compared to an experiment in which only PEDOT:PSS aggregates and no NPs were added to the inoculum. Mean current density after 40 h reached 3.58 ± 0.91 μA cm^−2^, compared to 0.16 ± 0.037 μA cm^−2^ in the control experiment. Taking a closer look at the improvement factor for steady-state current density (Fig. 7c), the addition of only NPs without a magnet beneath the anode (II) as well as the addition of PEDOT:PSS aggregates without NPs (III) does not have a significant influence, whereas the addition of magnetic, conductive NPs and the subsequent formation of micropillars on the anode surface (Fig. 7c–IV) significantly improves steady-state current density by a factor of 4.52 ± 1.06. The combination of PEDOT:PSS aggregates and magnetic, conductive micropillars leads to an even higher significant improvement factor of 21.89 ± 7.41 (Fig. 7c–V).

## 4 Discussion

### 4.1 Enhancement of electroactive surface area via conductive, magnetic nanoparticles

As displayed in Fig. 2, the utilization of magnetic, conductive NPs as building blocks for the formation of micropillar extensions leads to an increased anodic surface area. OCT analysis revealed an increase by approximately factor 12 for the chosen experimental conditions. By adjusting NP concentration, flow rate, magnetic field, etc. also different extension factors are likely to be achieved. Additionally, the assembled micropillar-structured electrode was investigated electrochemically by CV and compared to an untreated flat anode surface (Fig. 3). This experiment revealed a twofold increased electroactive surface area, calculated via Randles-Sevcik equation. First, this indicates, that the created micropillar structures are indeed electrically conductive and do improve the available electroactive surface area. If the added NPs were not electrically connected to the anode, then an even lower current density compared to the control experiment were to be expected, since the formed structures would then block the anode surface. Since the opposite is the case (higher peak current in CV after micropillar formation), we can be certain that the NPs indeed enhance the electroactive surface area.

The difference in surface area determined via OCT and CV might be due to several reasons. One factor is, that when performing CV, the observed current is dictated by diffusion of electrolyte towards the electrode, creating a diffusion layer around the electrode surface. Therefore, CV has its limitations when analyzing 3D electrodes and measures mainly the area of the outer contour of the diffusion layer and fails to capture smaller voids within the electrode structure [60]. This means, that the actual electroactive surface area is most probably higher, than determined via CV. Nonetheless, this technique is still useful, since it allows to make a qualitative statement about the increase of the electroactive surface area. Besides, it is also possible that surface area determined via OCT analysis is not electroactive throughout and there might be micropillar regions that are measurable via OCT, but these do not necessarily have to be electrically connected to the electrode. Taken together, it is difficult to exactly determine the increased electroactive surface area, but the two values generated via OCT and CV probably mark the range of the actual increase in electroactive surface area.

### 4.2 Nanoparticle agglomerates act as steerable carriers for microorganisms

Subsequently, the NP agglomerates were tested on their cytotoxicity, via a cell viability assay with the electroactive microorganisms *S. oneidensis* and *G. sulfurreducens*. Both species adhere spontaneously onto the agglomerates and show high viabilities with 79 % and 90 % living cells, respectively (Fig. 4). Since the particles are conductive and magnetic and do not show a substantial cytotoxicity, they are especially well suited as steerable carriers for electroactive microorganisms. In this case, the target would be an electrode, but the nanoparticle-microorganism-hybrids could be directed onto any surface of interest, thus allowing a fast and controlled formation of biofilms in any desired location. The only necessity is the presence of a magnetic field, so either a magnet needs to be placed behind the surface of interest or the surface itself is a magnet.

Our results from the abiotic experiments utilizing a movable magnet or a second magnet clearly demonstrate that the particles can be repositioned after their initial assembly on demand. However, it will be essential to develop a larger infrastructure surrounding the microfluidic bioelectrochemical system (BES) to integrate either electromagnets that can be turned on and off or larger permanent magnets, enabling biotic experiments that involve more significant structural changes to the nanoparticle architecture.

### 4.3 Micropillars improve performance of *S. oneidensis* and *G. sulfurreducens* biofilms

Consequently, the nanoparticle-microorganism-hybrids were inoculated into a microfluidic flow-through BES to investigate their effect on current density production. *S. oneidensis*, which is known to form only thin biofilms on anodes [22,61], was significantly improved in its ability to produce current by the formation of the micropillar-structured anode surface, but not when NPs were added without a magnet placed beneath the anode as anchoring and structuring feature. This leads to the conclusion that only a combination of magnetic, conductive NPs and an externally applied magnetic field results in the almost 5-fold improved performance of the biofilm. Assuming that the organisms can also form a biofilm monolayer on the conductive micropillars, this value is in line with the determined increase in electroactive surface area of the anode. To further overcome the organisms’ limited capability of anodic biofilm formation, PEDOT:PSS aggregates were employed and inoculated together with NPs and *S. oneidensis*. PEDOT:PSS was to serve as synthetic, conductive biofilm matrix, accommodating the bacteria not only directly on the anode or micropillar surface, but also within the PEDOT:PSS agglomerates entangled within the micropillars. The magnetically steered formation of this three-dimensional semi-synthetic biofilm led to a significant steady-state current density improvement factor of 21.89 ± 7.41 compared to the control. When only PEDOT:PSS aggregates where added to the *S. oneidensis* inoculum, no significant change in steady-state current density was observed compared to the control. The reason for this is probably that there is no tensile force in a flow-through system to hold the PEDOT:PSS aggregates on the anode. The combination with magnetic conductive NPs, on the other hand, leads to entrapment of PEDOT:PSS aggregates that are directly connected to the anode surface.

Therefore, the combination of both agents leads to a further increase in electroactive surface area, with the PEDOT:PSS aggregates apparently harboring a considerable amount of *S. oneidensis* cells. These results are in line with the work presented by Zajdel et al. [57], where a PEDOT:PSS based multilayer composite biofilm was electropolymerized on an anode, leading to a 20-fold increase in current density. Our smaller building blocks consist of magnetic, conductive NPs and pre-polymerized PEDOT:PSS aggregates and offer potential for higher versatility and reusability, since it is possible to remove, rearrange and collect these agents, by employing a suitable magnetic field. The prospect of magnetically tunable biofilms could additionally aid in overcoming prevailing bottlenecks as substrate limitation by steered reorganization of the microorganism-NP hybrids on the anode surface. Future studies in this field might focus on such tunable biofilms and their effects on productivity.

In a more recent study, Tseng et al. [59] achieved up to 178-fold increase in current density with a more complex coating of an indium tin oxide (ITO) electrode, consisting not only of PEDOT:PSS, but also crosslinked with poly(2-hydroxyethylacrylate) (PHEA) along with a layer of polydopamine. Here, the improvement factor is difficult to compare to our results, since ITO is a very smooth material, generally impeding biofilm formation, compared to rough carbon electrodes. It is, however, conceivable that coating the NPs used in our study with the approach used by Tseng and colleagues might further increase the produced current density, although this would entail a more complex implementation.

In case of *G. sulfurreducens* the micropillar structures on the anode did not lead to a significant change in steady-state current density compared to the control. For *Geobacter* the anode architecture does not seem to play a crucial role and surface roughness of the electrode does hardly affect steady-state current density for this organism. A study focusing on different anode materials for *G. sulfurreducens* by Kipf et al. [62] was leading to the same conclusion. On the other hand, in a study by Champigneux et al. [63] an increase in current density for *G. sulfurreducens* was achieved by nano/micropatterning gold electrodes with gold micropillars of 100 × 100 μm cross-section, 500 μm height and spacing of 100–200 μm. This, compared to our study, bigger spacing allowed for better substrate diffusion, which seems to be the limiting factor for improving current production of the thicker *Geobacter* biofilms. Therefore, less densely packed micropillars seem to be beneficial for improving the performance of this organism, while for *S. oneidensis* the opposite seems to be the case.

However, in our experiments with *G. sulfurreducens* the steady-state current density was reached after 18 h, compared to 72 h, respectively. This 4-fold increase in startup time is probably attributable to the fact, that the conductive, magnetic NPs act as steerable carriers and are actively pulled onto the anode surface with a magnet, eventually leading to an increased number of microorganisms immobilized on the anode surface directly upon inoculation and subsequently also leading to an improved startup time. According to Champigneux et al. [63], roughening the surface of the electrode surface also seems to play a role in decreasing the start-up time. This substantial decrease in startup time is especially advantageous for potential applications in process engineering, where delays in time lead to decreased productivity and therefore loss of value in the process.

Future work on this subject could also aim at improving the benefit gained by the applied magnetic field. In this study, we utilized a permanent magnet, replacing it with an electromagnet, would further increase controllability and versatility. By, for example, applying a spatially and temporally varying pattern of magnetic fields, the conductive magnetic micropillars could undergo permanent restructuring, potentially enhancing substrate diffusion towards the electrode surface. Comparable achievements were made by Demirörs et al. [64], who managed to produce soft magnetic carpets and control droplets of liquids with a changing magnetic field.

## 5 Conclusion

In this study, a recently described platform for microfluidic biofilm cultivation and analysis [22] was employed to investigate the formation of conductive, magnetic micropillars assembled by conductive, magnetic NPs on an electrode surface, as well as their impact on anodic biofilms of *S. oneidensis* and *G. sulfurreducens*. Assessment of NP cytotoxicity revealed negligible effects, and application of magnetic, conductive NPs resulted in enhanced performance in a BES for both organisms. Specifically, for *S. oneidensis* this led to a significant nearly 5-fold improvement in steady-state current density, which was further improved by combining with PEDOT:PSS aggregates to almost 22-fold. Despite this substantial enhancement, the steady-state current density remained around 1.6 % compared to *G. sulfurreducens*, indicating potential for further optimization to enhance the productivity of anodic *S. oneidensis* biofilms. In the case of *G. sulfurreducens* a 4-fold increase in startup time but no increase in steady-state current density was observed, when utilizing the conductive, magnetic NPs as steerable carriers.

Overall, these findings indicate that magnetic, conductive NPs can act as cost-effective and easy-to-use upgrade of already existing BES, which only requires a magnetic field at the electrode of interest. This procedure likely holds promise for transfer to other electroactive microorganisms, that form biofilms on electrodes, including possible applications in the emerging field of electrosynthesis.

## CRediT authorship contribution statement

**René Wurst:** Writing – review & editing, Writing – original draft, Visualization, Validation, Methodology, Investigation, Formal analysis, Data curation, Conceptualization. **Edina Klein:** Writing – review & editing, Visualization, Formal analysis, Data curation. **Johannes Gescher:** Writing – review & editing, Writing – original draft, Validation, Supervision, Resources, Project administration, Funding acquisition, Conceptualization.

## Declaration of competing interest

The authors declare the following financial interests/personal relationships which may be considered as potential competing interests:

Johannes Gescher reports financial support was provided by 10.13039/501100001659German Research Foundation. If there are other authors, they declare that they have no known competing financial interests or personal relationships that could have appeared to influence the work reported in this paper.

## Data Availability

Data will be made available on request.
